# Modulation of Cardiac Autonomic Function by Fingolimod Initiation and Predictors for Fingolimod Induced Bradycardia in Patients with Multiple Sclerosis

**DOI:** 10.3389/fnins.2017.00540

**Published:** 2017-10-12

**Authors:** Kai Li, Urszula Konofalska, Katja Akgün, Manja Reimann, Heinz Rüdiger, Rocco Haase, Tjalf Ziemssen

**Affiliations:** ^1^Autonomic and Neuroendocrinological Lab, Center of Clinical Neuroscience, University Hospital Carl Gustav Carus, Dresden University of Technology, Dresden, Germany; ^2^Department of Neurology, Beijing Hospital, National Center of Gerontology, Beijing, China; ^3^MS Center, Center of Clinical Neuroscience, University Hospital Carl Gustav Carus, Dresden University of Technology, Dresden, Germany

**Keywords:** fingolimod, multiple sclerosis, cardiac autonomic function, multiple trigonometric regressive spectral analysis, bradycardia, heart rate variability (HRV)

## Abstract

**Objective:** It is well-known that initiation of fingolimod induces a transient decrease of heart rate. However, the underlying cardiac autonomic regulation is poorly understood. We aimed to investigate the changes of autonomic activity caused by the first dose of fingolimod using a long-term multiple trigonometric spectral analysis for the first time. In addition, we sought to use the continuous Holter ECG recording to find predictors for fingolimod induced bradycardia.

**Methods:** Seventy-eight patients with relapsing-remitting multiple sclerosis (RRMS) were included. As a part of the START study (NCT01585298), continuous electrocardiogram was recorded before fingolimod initiation, and until no <6 h post medication. Time domain and frequency domain heart rate variability (HRV) parameters were computed hourly to assess cardiac autonomic regulation. A long-term multiple trigonometric regressive spectral (MTRS) analysis was applied on successive 1-h-length electrocardiogram recordings. Decision tree analysis was used to find predictors for bradycardia following fingolimod initiation.

**Results:** Most of the HRV parameters representing parasympathetic activities began to increase since the second hour after fingolimod administration. These changes of autonomic regulations were in accordance with the decline of heart rate. Baseline heart rate was highly correlated with nadir heart rate, and was the only significant predicting factor for fingolimod induced bradycardia among various demographic, clinical and cardiovascular variables in the decision tree analysis.

**Conclusions:** The first dose application of fingolimod enhances the cardiac parasympathetic activity during the first 6 h post medication, which might be the underlying autonomic mechanism of reduced heart rate. Baseline heart rate is a powerful predictor for bradycardia caused by fingolimod.

## Introduction

Fingolimod is highly effective for the treatment of relapsing-remitting multiple sclerosis (RRMS; Cohen et al., 2010; Calabresi et al., 2014). Its therapeutic effect is mediated by the modulation of sphingosine-1-phosphate (S1P) receptors in the lymphocytes and neural cells. Besides, S1P receptors also exist in cardiac myocytes (Brinkmann et al., 2010; Camm et al., 2014). Therefore, the first dose of fingolimod can lead to a transient decrease of heart rate (HR), which has been a major concern in the first years after its approval (Camm et al., 2014). A lot of clinical data have proven the cardiac safety of fingolimod in the real world until now (Limmroth et al., 2017; Ziemssen et al., 2017). That is why a specific first dose monitoring procedure should be included in the risk-management plan of fingolimod (Thomas et al., 2017).

HR is regulated by the autonomic nervous system. Spectral analysis of heart rate variability (HRV) is a valuable tool for the quantitative evaluation of the fluctuations of HR, and can reflect the parasympathetic activities of cardiac autonomic function (Task Force of the European Society of Cardiology and the North American Society of Pacing and Electrophysiology, 1996). Unlike the well-known HR change caused by fingolimod, the alteration of cardiovascular autonomic function following fingolimod initiation has been less described. Recently, Simula et al. (2017) and Hilz et al. (2017) reported the sequence of cardiovascular autonomic parameter changes following fingolimod initiation. These two studies showed that HRV parameters representing parasympathetic modulation increased after fingolimod initiation, but there are some inconsistencies between them and both studies enrolled a relatively small sample of patients with RRMS. It should be noted that they applied different approaches, Simula et al. employed an hourly spectral analysis with fast Fourier transform on continuous ECG recordings of several hours and Hilz et al. utilized a spectral analysis with trigonometric regressive spectral (TRS) analysis on short ECG and beat-to-beat blood pressure recording segments of 2 min.

Most commonly, a power spectral analysis of HRV is performed through the fast Fourier transform, which requires equal distance between adjacent heart beats (RR interval, RRI). However, RRIs are non-equidistant and therefore interpolation is needed. The interpolation for the fast Fourier transform can affect the accuracy of spectral analysis (Rudiger et al., 1999; Ziemssen et al., 2008, 2013). Unlike the fast Fourier transform, TRS analysis does not need interpolation on non-equidistant RRIs. Alternatively, it analyzes the original RRI and blood pressure data and obtains the spectral components more accurately by using a trigonometric regression (Rudiger et al., 1999; Ziemssen et al., 2013). Its excellent performance has been verified by the EuroBaVar study (Laude et al., 2004). TRS analysis has not been employed in the analyses of the hourly HRV changes following the first dose of fingolimod using continuous Holter ECG recordings so far. Furthermore, TRS analysis is traditionally applied on the manually chosen 1–2 min electrocardiogram (ECG) segments (Reimann et al., 2010, 2012). In the present study, we are the first to use a long-term spectral analysis technique via TRS to calculate hourly HRV parameters using Holter ECG recordings. To achieve this aim, we developed a strategy of analyzing all the successive 1-min-length ECG segments of the entire ECG time window, and then we can generate the hourly averaged HRV parameters. This approach would make full use of the recorded continuous ECG information and yield more stable results.

In clinical practice, a predictor for fingolimod induced bradycardia would help physicians select appropriate patients and decide proper monitoring strategies. Several studies explored the relationship between autonomic function testing before fingolimod administration and the fingolimod-induced HR response. Simula et al. (2016) demonstrated that the percentage of successive normal RR intervals differing by >50 ms (pNN50) calculated by the 24-h ECG recording at baseline was associated with HR decline caused by fingolimod. Rossi and colleagues revealed the correlation between Valsalva ratio, HR variation in metronomic deep breathing and bradycardia resulted from fingolimod (Rossi et al., 2015). Hilz et al. (2015) demonstrated that a comprehensive autonomic battery could detect autonomic dysfunction in patients with delayed HR recovery after fingolimod intake. Although these studies used a small sample size, they suggested that HRV might be promising in predicting fingolimod induced HR decline. But their approaches require a professional autonomic testing laboratory or a 24-h ECG recording before medication. A continuous ECG monitoring after the first dose of fingolimod is recommended by the European Medicine Agency (EMA) and commonly used in clinical practice. We assumed that the HRV parameters obtained from continuous ECG might facilitate the identification of patients with high risk for bradycardia after fingolimod initiation, and might provide a convenient predictor for daily clinical use (Thomas et al., 2017).

This study sought to demonstrate the change of HRV parameters within 6 h after fingolimod intake, and tried to find convenient predictors for bradycardia induced by fingolimod in a relatively large patient sample.

## Methods

### Participants

Eighty-one patients with RRMS were consecutively included in this study as a Dresden sub-study of the multicenter START study (NCT01585298; Limmroth et al., 2017). The diagnosis of RRMS was based on the McDonald criteria (Polman et al., 2011). Fingolimod was prescribed at a daily dose of 0.5 mg according to the label set by the EMA. The patients were either with highly disease activity despite an adequate treatment with at least one disease modifying therapy, or with rapidly evolving severe RRMS. Only patients older than 18 years were enrolled. Exclusion criteria included: using antiarrhythmic medication or other drugs which may reduce HR; with a history of second degree Mobitz Type II or higher-degree AV block, sick-sinus syndrome, sinoatrial heart block, significant QT prolongation, symptomatic bradycardia or recurrent syncope; with known ischemic heart disease, cerebrovascular disease, myocardial infarction, hypokalemia, congestive heart failure, cardiac arrest, uncontrolled hypertension, or severe sleep apnea. Demographical and clinical information including the Expanded Disability Status Scale (EDSS) scores of the patients was collected.

The study was in accordance with relevant guidelines and regulations, and approved by the Institutional Review Board of University Hospital Carl Gustav Carus. This study was carried out according to the Declaration of Helsinki. All the subjects gave written informed consent prior to participation.

### Study procedures

Fingolimod was administered before 10:00 in the morning. The patients' brachial blood pressure (using the mercury sphygmomanometer) and HR were measured before medication and every hour for at least 6 h after medication. Continuous 12-lead ECG recording was performed using CardioMed CM3000-12 (Getemed, Teltow, Germany) since 1 h before medication, and until no <6 h post medication. In general, the patients were in a sedentary state in the waiting room of our hospital during the ECG monitoring, although their physical activity was not restricted and they breathed freely. All the ECG recordings were checked by a cardiology expert for any abnormalities.

### Heart rate variability analysis

Mean HR was computed from mean RRI of each hour, and bradycardia was defined as a HR < 60 bpm (Camm et al., 2014). Time domain parameters including the standard deviation of normal RR intervals (SDNN), the root mean square of successive differences of RR-intervals (RMSSD), and pNN50 were calculated hourly according to well-accepted standards (Task Force of the European Society of Cardiology and the North American Society of Pacing and Electrophysiology, 1996). The frequency domain parameters were computed by the long-term multiple trigonometric regressive spectral (MTRS) analysis.

In general, TRS analysis is working with a single time window (local data segments) in the range of 20–60 s. The regression function in TRS has a general form: Reg (t) = A*sin(ωt+ψ); A is the amplitude, ωt is the angular frequency, and ψ is the phase shift of the trigonometric regression function Reg (t). To determine the function Reg (t), we need to make the deviations of Reg (t) from the original values minimal: F = Σ (RRI (t) –Reg (t))2 = Min and /or dF = 0. By pursuing a minimal deviation of Reg (t), we can determine the variables A, ω, and ψ with the help of partial differential quotients through the following equations: dF/dA = 0; df/dω = 0; df/d ψ = 0. Then optimal oscillations can be found by variation of the frequency for a maximal adaption to the original RR intervals (Rudiger et al., 1999; Ziemssen et al., 2013).

By shifting these local data segments with one, two or more beats, it is possible to measure the time variability over a global ECG data segment of 1–3 min (consisting of *multiple* local data segments). In this case TRS analysis is called MTRS analysis. This procedure guarantees a statistically representative spectral analysis for the global data segment (Rudiger et al., 1999; Ziemssen et al., 2013). Traditionally, we manually chose a stable segment of 1–2 min as the global data segment, within which local data segments of 30 s were analyzed and shifted beat by beat to determine the frequency domain parameters of the selected global data segment (Reimann et al., 2010, 2012). In the present study, we also used a local time window of 30 s, and this was shifted beat by beat within a global data segment of 1 min. After that, spectral results obtained from these global 1-min segments (60 global segments in an hour) were averaged over the entire hour. Artifacts and extrasystoles were manually identified and corrected. Low frequency power of HRV (LF), and high frequency power of HRV (HF) were calculated through the long-term MTRS analysis. LF power is the spectral component between 0.04 and 0.15 Hz, and HF power is the spectral component between 0.15 and 0.4 Hz. LF and HF were expressed as absolute values, as well as relative values which were the proportions (in percent) of LF and HF powers in the total power. HF represents mainly the parasympathetic cardiovagal tone, while there are controversies in the interpretation of LF. The Task Force of the European Society of Cardiology and the North American Society of Pacing and Electrophysiology (1996) considered LF as a parameter that included both sympathetic and vagal influences, while some researchers disagree with this opinion (Reyes del Paso et al., 2013).

### Statistical analysis

All statistical analyses were performed using SPSS for Windows (Version 23.0. Armonk, NY: IBM Corp). Data are presented as mean ± standard error of the mean (SEM) unless stated otherwise. Normality of variables was assessed by the Kolmogorov–Smirnov test. Logarithmic (ln) transformation was used in right-skewed variables if applicable. Changes of the measurements across different time points were tested by the repeated measures ANOVA. Greenhouse-Geisser correctional adjustment was applied if the assumption of sphericity was violated. To explore potential modifying factors, sex was included as a between-subjects factor, while age and EDSS were added as covariates. For variables with a non-normal distribution, Friedman test was performed to assess the changes between different time points. *Post-hoc* analyses were adjusted by the Bonferroni method.

Based on the data distribution, Pearson (normal) or Spearman's (non-normal) correlation was used to explore the associations between demographic and clinical variables, cardiovascular parameters and the nadir HR. Decision tree analysis is a powerful statistical tool for prediction, and can effectively subdivide continuous variables into subgroups (Song and Ying, 2015). The exhaustive Chi-squared automatic interaction detection method (CHAID) was utilized. Nadir HR after fingolimod intake and the categorical variable whether the patient had bradycardia were used as the dependent variables. The independent variables were demographic and clinical information, and cardiovascular parameters prior to fingolimod intake. A 10-fold cross-validation was adopted. The maximum tree depth was set as three levels, the minimum parent and child node sizes were 10 and five, respectively. The significance level for splitting was 0.05. Bonferroni correction was applied during the decision tree analysis. For all the above analyses, results were considered statistically significant when *p* < 0.05.

## Results

### Study population

Three of the recruited patients lacked part of the required time window of continuous ECG monitoring and were excluded for further analyses. Finally, 78 patients with RRMS were included for the analyses. Their demographic and clinical characteristics are shown in Table 1. The mean (SEM) age of the male and female patients were 38.7 (2.2) and 39.9 (1.6) years, and there was no significant difference between their ages (*t* = −0.442, *p* = 0.66). One patient underwent an extended monitoring of 2 h due to delayed HR recovery. There was no rebound arrhythmia in the included participants. All the patients' concomitant diseases were in a stable state. Patients with depression were mainly treated by selective serotonin reuptake inhibitors or serotonin norepinephrine reuptake inhibitors. Three patients with hypertension underwent angiotensin-converting enzyme inhibitor monotherapy, one hypertensive patient took Ramipril and hydrochlorothiazide, and the other hypertensive patient took telmisartan and amlodipine. The patients diagnosed with hypothyroidism were treated with L-thyroxin. There was no patient with diabetes mellitus.

**Table 1:** Demographic and clinical features of the participants.

Number of patients	78
Sex (female/male)	47/31
Age (years)	39.4 ± 1.3
Disease duration since RRMS diagnosis (years)	6.9 ± 0.6
EDSS	3.1 ± 0.2
Height (cm)	172.1 ± 1.1
Weight (kg)	73.5 ± 1.7
Concomitant diseases (number of patients)^*^	
Depression	8
Hypertension	5
Hypothyroidism	5

*Values are presented as mean ± SEM*.

* *The concomitant diseases which occurred in at least two patients are displayed. EDSS, Expanded Disability Status Scale; RRMS: relapsing-remitting multiple sclerosis*.

### Changes of cardiovascular parameters after fingolimod administration

**Figure 1:**
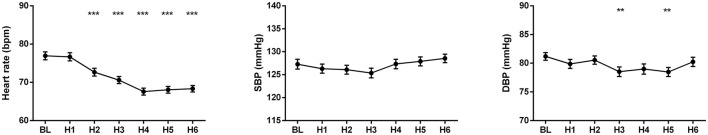
Changes of heart rate and blood pressure after fingolimod administration. BL, baseline; DBP, diastolic blood pressure; H1, H2, H3, H4, H5, H6, the first, second, third, fourth, fifth, and sixth hour after fingolimod intake; SBP, systolic blood pressure. Values are presented as mean ± SEM. ^**^p < 0.01 compared with baseline, ^***^p < 0.001 compared with baseline.

**Figure 2:**
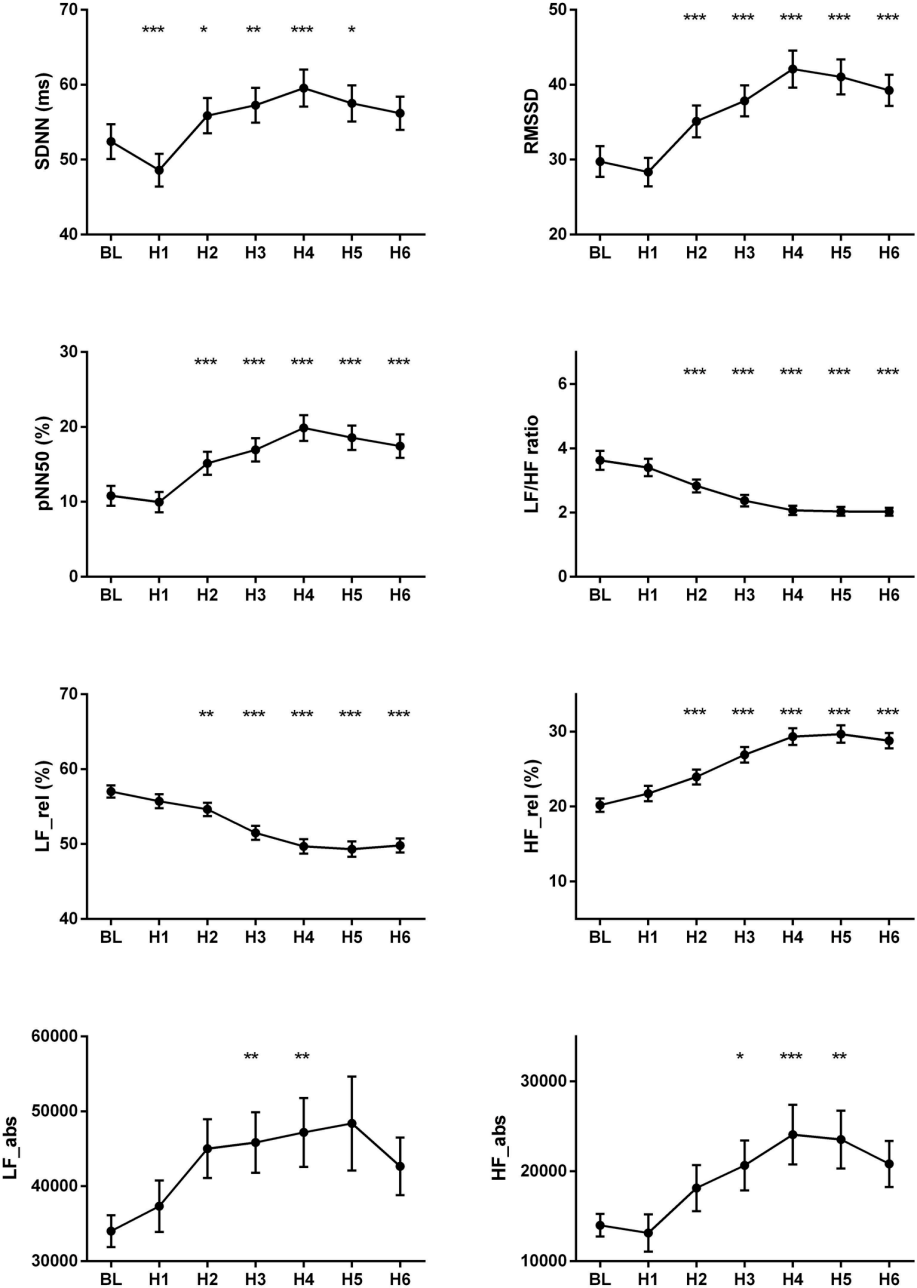
Changes of the cardiovascular parameters after fingolimod administration. BL, baseline; H1, H2, H3, H4, H5, H6, the first, second, third, fourth, fifth, and sixth hour after fingolimod intake; HF_abs, absolute value of high frequency power; HF_rel, relative value of high frequency power; LF_abs, absolute value of low frequency power; LF_rel, relative value of low frequency power; pNN50, percentage of successive normal RR intervals differing by >50 ms; RMSSD, the root mean square of successive differences of RR-intervals; SDNN, the standard deviation of normal RR intervals. Values are presented as mean ± SEM. ^*^p < 0.05 compared with baseline, ^**^p < 0.01 compared with baseline, ^***^p < 0.001 compared with baseline.

**Figure 3:**
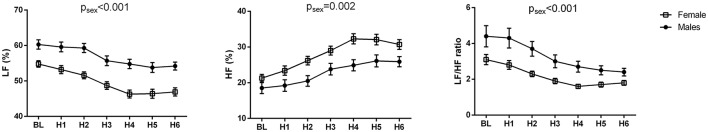
Change of frequency domain parameters in male and female patients after fingolimod administration. BL, baseline; H1, H2, H3, H4, H5, H6, the first, second, third, fourth, fifth, and sixth hour after fingolimod intake; HF, relative value of high frequency power; LF, relative value of low frequency power. Values are presented as mean ± SEM. *p*_sex_, the significance of sex as a main effect.

The changes of HR, systolic and diastolic blood pressure, SDNN, pNN50, RMSSD, absolute and relative values of LF and HF powers, and LF/HF ratio were presented in Figures 1, 2. The mean HR was lowest at the fourth hour after medication, and the nadir HR was 65.4 ± 0.8 bpm. Bradycardia (HR < 60 bpm) appeared in 19 patents. The decrease of HR from baseline to nadir was 11.5 ± 0.7 bpm. Except for SDNN, blood pressure, absolute values of LF and HF powers, all the other variables showed a consistent trend: the value deviated from baseline since the second hour after fingolimod intake, arrived at their peaks or nadirs at the fourth, fifth, or sixth hour after medication. Although the time points of their peaks/nadirs were not exactly the same, the pairwise comparisons between the fourth, fifth and sixth hours obtained non-significant results in all these parameters. SDNN decreased at the first hour post medication, and then began to increase since the second hour after fingolimod initiation. *Post-hoc* pairwise comparison did not reveal a significant decrease of systolic blood pressure after medication, while diastolic blood pressure during the third and fifth hour was significantly lower than baseline. Absolute LF power increased after fingolimod initiation, with a significant difference from baseline at the third and fourth hour. Absolute HF power also increased after fingolimod administration, with a significant difference from baseline at the third, fourth, and fifth hour. Sex, age, and EDSS had no significant interaction with the effect of fingolimod in these parameters. Sex had a significant main effect in frequency domain parameters, female patients had a lower relative value of LF power and LF/HF ratio than male patients (all *p* < 0.001), and a higher relative value of HF than male patients (*p* = 0.002; Figure 3).

### Correlation analysis

The results of the correlation analyses between nadir HR during the 6 h after fingolimod administration and various demographic, clinical, and cardiovascular parameters are shown in Table 2. Age, weight, baseline HR, and time domain parameters were significantly correlated with nadir HR. In particular, baseline HR had the strongest association with the nadir HR, while other variables had only weak associations.

**Table 2:** Correlation analysis between demographic, clinical, and cardiovascular parameters and nadir heart rate.

	**Correlation coefficient with nadir heart rate**	***p*-value**
Age	−**0.249**	**0.028**
Weight	−**0.238**	**0.036**
BMI	−0.151	0.188
EDSS^*^	−0.063	0.581
Disease duration since RRMS diagnosis^*^	0.014	0.903
Baseline heart rate	**0.709**	**<0.001**
Baseline SBP	−0.084	0.465
Baseline DBP	0.148	0.197
Baseline SDNN	−**0.354**	**0.001**
Baseline RMSSD	−**0.333**	**0.003**
Baseline pNN50	−**0.288**	**0.010**
Baseline LF	0.179	0.118
Baseline HF	−0.168	0.141
Baseline LF/HF ratio	0.157	0.170

* *For EDSS and disease duration since RRMS diagnosis, Spearman correlation was employed. For other variables, Pearson correlation was adopted. BMI, body mass index; DBP, diastolic blood pressure; EDSS, Expanded Disability Status Scale; HF, relative value of high frequency power; LF, relative value of low frequency power; pNN50, percentage of successive normal RR intervals differing by >50 ms; RMSSD, the root mean square of successive differences of RR-intervals; RRMS, relapsing-remitting multiple sclerosis; SBP, systolic blood pressure; SDNN, the standard deviation of normal RR intervals. Significant correlations are shown in bold font*.

### Decision tree analysis

**Figure 4:**
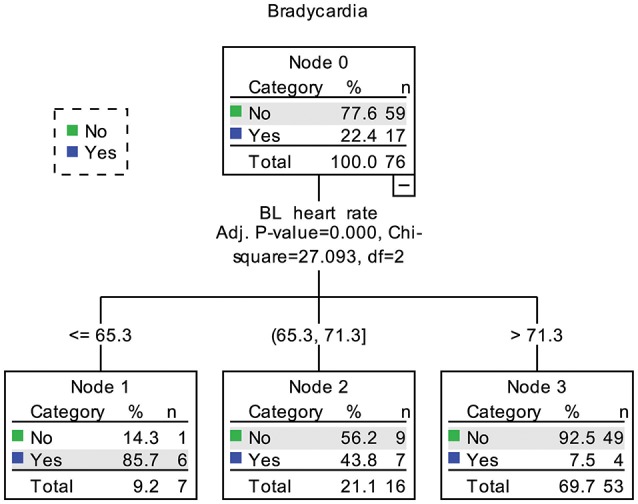
Decision tree of predictors for bradycardia caused by fingolimod. BL, baseline. In node 1, 2 patients already had bradycardia before fingolimod intake were not included.

In the decision tree analysis, the demographic, clinical, and cardiovascular variables in Table 2 were used as independent variables. Because the aim was to find predicting factors for fingolimod induced bradycardia, two patients with a baseline HR lower than 60 bpm were excluded. The models using nadir HR after fingolimod intake and whether the patient had bradycardia caused by fingolimod obtained similar results. Baseline HR was the only significant predicting factor (Figure 4). According to baseline HR, the patients were divided into three groups: 85.7% of the patients with a baseline HR < 65.3 bpm had bradycardia during post medication monitoring, 43.8% of the patients with a baseline HR between 65.3 and 71.3 bpm had bradycardia, and only 7.5% of the patients with a baseline HR higher than 71.3 had bradycardia within 6 h after fingolimod initiation.

## Discussion

This is the first study which implemented long-term MTRS analysis in successive 1-h-length ECG segments analysis. Our results disclosed the consistent changes of HR and HRV parameters after fingolimod initiation, and indicated that baseline HR was the strongest predictor for nadir HR among various cardiovascular parameters.

Because the changes of cardiac parameters after fingolimod intake is a dynamic process over several hours, manually choosing or recording a several-minute-length ECG segment from each hour for analysis might be affected by subjectivity of the selection and inconsistency of the exact position of chosen segments between patients, thus could increase systematic bias. This is also the case for delineating slower alterations such as circadian changes of cardiac autonomic regulation (Parati et al., 1990; Bilan et al., 2005). Automatically calculating the hourly HR and HRV parameters can solve these problems. Previous long-term spectral analyses of HRV were mainly performed with the fast Fourier transform. The most commonly used time windows for long-term spectral analyses of HRV were 1 and 24 h. There has been two main approaches: viewing the target time window as a whole data segment or averaging the spectral results of all the shorter segments (e.g., 2 min) within the whole time window. Generally, these two approaches yield similar results for LF and HF power components and the averaging process is more popular (Rottman et al., 1990; Task Force of the European Society of Cardiology and the North American Society of Pacing and Electrophysiology, 1996; Kleiger et al., 2005). In addition, long-term spectral analysis especially for 24 h can be used to evaluate cardiac autonomic regulations during normal daily activities, and is valuable for prognosis prediction (Task Force of the European Society of Cardiology and the North American Society of Pacing and Electrophysiology, 1996; Kleiger et al., 2005). To some extent, the comparison between 24-h long-term and short-term spectral analysis is similar as the comparison between 24-h ambulatory and clinic blood pressure measurements (Chobanian et al., 2003). However, there are some shortcomings of the long-term spectral analysis. Cardiac autonomic regulations underlying LF and HF power components cannot be deemed as stationary in long-term spectral analysis. Additionally, the averaging process integrate the dynamic alterations of autonomic tone within the specified time window, which prevents assessing autonomic function fluctuations in this time widow. Nevertheless, long-term spectral analysis have been widely applied and considered useful in evaluating cardiac autonomic function changes in various conditions and risk stratifications in cardiovascular diseases (Pagani et al., 1988; Parati et al., 1990; Tsuji et al., 1994, 1996; Task Force of the European Society of Cardiology and the North American Society of Pacing and Electrophysiology, 1996; Rossinen et al., 1997; Bilan et al., 2005; Kleiger et al., 2005), In this study, we utilized the long-term MTRS analysis using a strategy that averaged the results of 60 one-minute global segments within an hour, and applied this long-term MTRS in estimating HRV change for the first time.

The present study showed that parameters reflecting parasympathetc function such as RMSSD, pNN50, and HF power increased after fingolimod intake, while HR, LF power and LF/HF ratio decreased after medication. This phenominan can be explained by the vagomimetic effect of fingolimod. During the first several hours after fingolimod administration, this medication activates the S1P G protein-gated potassium channels in the myocytes, which is a vagomimetic effect and similar to the action of acetylcholine on muscarinic receptors (Camm et al., 2014; Vanoli et al., 2014). This can explain our findings that parameters representing parasympathetic activities initially increased after fingolimod intake, and heart rate decreased accordingly. Then several hours later, the down-regulation of S1P receptors in the myocytes mediates the restoration of the cardiovascular autonomic function (Camm et al., 2014; Vanoli et al., 2014). This S1P receptor down-regulation accounts for the decline of parasympathetic HRV parameters since the fifth or the sixth hour after fingolimod intake in our study.

In the present study, heart rate and most of the HRV parameters showed a consistent deviation from baseline since the second hour after fingolimod application. This is incompatible with the study by Simula et al. (2017). In their study, the time points when RR interval and HRV parameters deviated from baseline varied. The significant change occurred at the first, second or third hour after fingolimod administration (Simula et al., 2017). In that study, only 27 patients were recruited, and *post-hoc* multiple pairwise comparisons were not corrected. Since we enrolled a much larger sample size, and used the Bonfferoni correction during pairwise comparisons to avoid false positivities, our results of this concordant change of the above cardiac parameters are more trustworthy.

Hilz et al. (2017) showed the changes of cardivascular autonomic parameters after fingolimod initiation also using TRS. In that study, they analyzed eight 2-min ECG and blood pressure data segments from baseline to 6 h after medication. In their study, RRI, SDNN, RMSSD began to deviate from baseline since the first hour after fingolimod adminiatration, and achieved their peak 5, 2, and 4 h after medication respectively. The absolute values of LF and HF both increased 1 h after fingolimod intake. Then the absolute value of HF continued to increase until its peak 4 h after medication, while the absolute value of LF underwent a transient significant increase 1 h after fingolimod intiation and then went back to the baseline value. In addition, the normalized values of LF and HF began to deviate from baseline since 3 h after medication, and achieved their peak/nadir 4 h after medication. The length of data analyzed affects time domain and frequency domain HRV parameters, especially SDNN is vulnerable to the influence of data length (Task Force of the European Society of Cardiology and the North American Society of Pacing and Electrophysiology, 1996). Although, the exact time points of changes of these paramters were not the same as our study, considering the methodological differences, the trends of changes were similar in RRI (HR in our study), RMSSD, LF/HF ratio of RRI, and LF and HF powers in our study and the study by Hilz et al. The absolute value of HF reflects parasympathetic activity and the absolute value of LF contains both parasympathetic and sympathetic regulations, this might partially explains the increase of absolute LF power after fingolimod intake in both studies (due to the increase of its parasympathetic component).

It is noteworthy that the decrease of SDNN in the first hour after fingolimod intake was not consistent with other parameters in the present study. In the study by Simula et al. (2017), SDNN was similar to baseline in the first hour after medication, while other time domain HRV parameters consistently increased since the first hour after fingolimod intake. In the study by Hilz et al., SDNN increased 1 h after medication together with RRI and RMSSD, but the time points of their peaks were different. One of the reason of this inconsistency is that SDNN represents all the cyclic components responsible for variability of analyzed RRI (Task Force of the European Society of Cardiology and the North American Society of Pacing and Electrophysiology, 1996), its underlying mechanism is different from the other cardiovascular autonomic parameters in our study and the study by Simula et al. Another reason of this inconsistency change of SDNN during the first hour post medication might be the emotional excitement of receiving a new oral treatment for their disease. It is reported that emotional change could affect HRV parameters (Pagani et al., 1989; Appelhans and Luecken, 2006; Hilz et al., 2017). Whether and how emotional response to the initiation of fingolimod affect cardiovascular autonomic function in patients with multiple sclerosis warrant further investigation.

The diastolic blood pressure decreased significantly in the third and fifth hour after fingolimod administration in our study. Previous studies including very large patient samples demonstrated that fingolimod could slightly decrease blood pressure in the first several hours (Camm et al., 2014). However, studies enrolling small patient samples reported inconsistent results (Hilz et al., 2017; Simula et al., 2017). It is common that a small sample size is not sensitive to detecting minor differences.

A previous study showed that pNN50 might predict the HR decrease caused by fingolimod (Simula et al., 2016). However, in our study, baseline HR was the strongest predictor for bradycardia caused by fingolimod. Time domain HRV parameters also had significant correlations with nadir HR, but their associations were much weaker than baseline HR. The results of decision tree analysis also underlined the importance of baseline HR, which was the only dominant predicting factor among various demographic, clinical and cardiovascular variables. Patients with a baseline HR higher than 71.3 bpm had the lowest risk for bradycardia. This result is reasonable, as the mean maximum decrease of HR due to fingolimod was 11.5 bpm in the present study. The mean maximum HR decrease in preceding studies was between 8 and 12 bpm (Cohen et al., 2010; Calabresi et al., 2014; Camm et al., 2014). It is also intuitive that patients with a lower baseline HR are at high risk for fingolimod induced bradycardia. Although our sample is not very large, and decision tree analysis might not be highly stable in this case (Song and Ying, 2015). Considering the significance of baseline HR in both the correlation and decision tree analyses, baseline HR is the most powerful predictor for nadir HR after fingolimod initiation in our study. In future studies trying to find predictors for finglimod induced bradycardia, we suggest that comparison between potential predictors with baseline HR should be performed. In clinical practice, we need to pay more attention to patients with a lower baseline HR bpm because of their higher risk for bradycardia.

In the present study, we found that female patients with RRMS had a higher relative value of HF, and a lower relative value of LF and LF/HF ratio than male patients. Thus, the female patients had higher parasympathetic activity compared with the male patients. As far as we know, our study is the first to report this sex difference in RRMS. It has been reported that healthy females had a higher HF, as well as lower LF and LF/HF ratio compared with healthy males. This gender difference in autonomic balance may be attributed to several factors: estrogen, oxytocin, and neuro controls (Koenig and Thayer, 2016). However, whether the mechanism underlying gender difference in RRMS is the same as that in the healthy subjects needs further investigation.

Our study has several limitations. Firstly, respiration was not controlled in the study, and the patients' activity was not strictly restricted. Although the patients were generally in a sedentary state and breathed peacefully, physical activity and breathing rate/tidal volume can potentially affect both time and frequency domain HRV parameters, and this could be a confounding factor in the analysis of the effects of fingolimod and gender on the HRV parameters (Yamamoto et al., 1991; Brown et al., 1993; Osterhues et al., 1997; Gąsior et al., 2016). Secondly, as we have mentioned in the Method section, there are controversies in the interpretation of LF and LF/HF ratio of heart rates. Whether they can reflect cardiac sympathetic activity is still unsure. We include the results of LF and LF/HF ratio for potential comparison with similar studies. Thirdly, continuous beat-to-beat blood pressure monitoring was not performed together with ECG monitoring. As LF oscillations of systolic blood pressure reflects sympathetically mediated peripheral vasomotor tone, this limitation restrains us from evaluating the vascular sympathetic modulation.

In conclusion, the present study showed that long-term MTRS was a useful tool for the determination of the dynamic change of HRV. Our findings demonstrated the consistent changes of cardiovascular parasympathetic activity and HR after fingolimod administration. Furthermore, among various demographic, clinical, and cardiovascular parameters, baseline HR was the strongest predictor for nadir HR after fingolimod initiation.

## Author contributions

Conception and design of the study: TZ, UK, KA. Recruiting the patients and collecting demographic and clinical information: TZ, UK, KA. Acquisition, analysis and interpretation of data: KL, RH, HR, MR. Drafting the manuscript: KL, TZ, all the other authors critically revised the draft and approved the final version.

### Conflict of interest statement

KA received personal compensation from Novartis, Biogen Idec, and Roche for the consulting service. TZ received personal compensation from Biogen Idec, Bayer, Novartis, Sanofi, Teva, and Synthon for the consulting services. TZ received additional financial support for the research activities from Bayer, Biogen Idec, Novartis, Teva, and Sanofi Aventis. UK received travel support by Teva. The other authors declare that the research was conducted in the absence of any commercial or financial relationships that could be construed as a potential conflict of interest.
